# The Increasing Burden of Imported Chronic Hepatitis B — United States, 1974–2008

**DOI:** 10.1371/journal.pone.0027717

**Published:** 2011-12-07

**Authors:** Tarissa Mitchell, Gregory L. Armstrong, Dale J. Hu, Annemarie Wasley, John A. Painter

**Affiliations:** 1 Immigrant, Refugee, and Migrant Health Branch, Centers for Disease Control and Prevention, Atlanta, Georgia, United States of America; 2 Division of Viral Diseases, Centers for Disease Control and Prevention, Atlanta, Georgia, United States of America; 3 Division of Viral Hepatitis, Centers for Disease Control and Prevention, Atlanta, Georgia, United States of America; 4 Global Immunization Division, Centers for Disease Control and Prevention, Atlanta, Georgia, United States of America; 5 Department of Pediatrics, Emory University School of Medicine, Atlanta, Georgia, United States of America; Broad Institute of Massachusetts Institute of Technology and Harvard University, United States of America

## Abstract

**Background:**

Without intervention, up to 25% of individuals chronically infected with hepatitis B virus (HBV) die of late complications, including cirrhosis and liver cancer. The United States, which in 1991 implemented a strategy to eliminate HBV transmission through universal immunization, is a country of low prevalence. Approximately 3,000–5,000 U.S.-acquired cases of chronic hepatitis B have occurred annually since 2001. Many more chronically infected persons migrate to the United States yearly from countries of higher prevalence. Although early identification of chronic HBV infection can reduce the likelihood of transmission and late complications, immigrants are not routinely screened for HBV infection during or after immigration.

**Methods:**

To estimate the number of imported cases of chronic hepatitis B, we multiplied country-specific prevalence estimates by the yearly number of immigrants from each country during 1974–2008.

**Results:**

During 1974–2008, 27.9 million immigrants entered the U.S. Sixty-three percent were born in countries of intermediate or high chronic hepatitis B prevalence (range 2%–31%). On average, an estimated 53,800 chronic hepatitis B cases were imported to the U.S. yearly from 2004 through 2008. The Philippines, China, and Vietnam contributed the most imported cases (13.4%, 12.5%, and 11.0%, respectively). Imported cases increased from an estimated low of 105,750 during the period 1974–1977 to a high of 268,800 in 2004–2008.

**Conclusions:**

Imported chronic hepatitis B cases account for approximately 95% of new U.S. cases. Earlier case identification and management of infected immigrants would strengthen the U.S. strategy to eliminate HBV transmission, and could delay disease progression and prevent some deaths among new Americans.

## Introduction

Hepatitis B virus infection, which is transmitted via blood or body fluids, is currently among the world's top ten causes of infectious disease-related mortality, resulting in over 500,000 deaths annually [1]. Acute infection with HBV is often asymptomatic, and the risk of progression to chronic infection is inversely related to age: approximately 90% of those infected before 1 year of age will develop chronic infection, while far fewer individuals infected after 5 years of age develop chronic infection [2]–[3]. Individuals with chronic hepatitis B may be a major source of HBV transmission, but are often unaware of their status [4].

Without intervention, approximately 25% of people with chronic infections acquired before age 5 and 15% of those acquiring infection at age 5 or after will die of late complications, such as cirrhosis and hepatocellular carcinoma [4]. In fact, >50% of all cases of hepatocellular carcinoma worldwide are associated with HBV [5]. Early identification of infected patients allows for the institution of medical monitoring and potentially therapy that could delay or prevent late complications [4].

In 1991, the United States implemented a comprehensive national strategy to eliminate HBV transmission through universal immunization. Subsequently, the estimated incidence of new, domestically acquired cases of chronic hepatitis B declined from approximately 10 cases/100,000 population in 1991 to the current rate of 1.2 cases/100,000 (CDC, unpublished data). The United States also has a low (∼0.3%) prevalence of chronic hepatitis B [6]. In contrast, over 80% of the world's population lives in countries of intermediate (2%–7%) or high (≥8%) prevalence [7]; although globally, hepatitis B (HB) vaccine programs have expanded in recent years, most programs are too new to have had an impact on the prevalence of chronic hepatitis B in adolescents and adults.

The United States receives approximately 1 million immigrants per year, mostly from countries of high or intermediate prevalence. The Centers for Disease Control and Prevention (CDC) recommends offering serologic screening for HBV infection to all foreign-born residents from countries of intermediate to high prevalence [4]. Serum hepatitis B surface antigen- (HBsAg) screening is a reliable and relatively inexpensive means to detect hepatitis B virus infection. Chronically infected immigrants could gain years of life if appropriate management is instituted before symptoms occur [4]. However, the extent to which screening recommendations for immigrants are followed is unknown.

The objectives of our analysis were to estimate the number of chronic hepatitis B cases imported to the United States during 1974–2008, to determine major regions and countries of origin for imported cases, to compare these numbers with the estimated number of new U.S.-acquired chronic hepatitis B cases over that time period, and to estimate mortality associated with imported chronic hepatitis B cases.

## Methods

We defined a case of imported chronic hepatitis B as pre-existing chronic hepatitis B in a person immigrating to the United States during 1974–2008. An immigrant was defined as any person obtaining U.S. legal permanent residence during those years. This definition excludes tourists, individuals on student or work visas, and undocumented immigrants, but includes legal immigrants who arrived from abroad after obtaining an immigration visa, as well as visitors, refugees, or asylees already in the United States who adjusted their legal residency status to that of “permanent resident” (obtained a Green Card) [8]. These applicants are required to undergo medical screening prior to immigration or at the time of adjustment of status [9].

We defined a case of U.S.-acquired chronic hepatitis B as chronic hepatitis B in an individual who was infected inside the United States.

### Data sources

Country-specific HBsAg prevalence rates were obtained from CDC and WHO data (CDC expert solicitation circa 2003, unpublished). Annual data on immigration by country and by age category were obtained from Department of Homeland Security (DHS) reports [10]. The number of new U.S.-acquired cases of chronic hepatitis B was modeled from the number of cases of acute hepatitis B reported to CDC through local and state health departments from 1980 through 2008 [11]. The first step of this model estimated the number of HBV infections each year by dividing the reported number of cases in each age group by the age-specific proportion of HBV infections that produce jaundice (ranging from 5% in newborns to 30% in adults, based on data from McMahon et al. [12]) and then multiplying by a constant to account for under-reporting. The value of this constant (2.8) was calibrated using an estimate of infections in persons less than 40 years old during the same period of time, obtained by means of “catalytic modeling” [13]. In the second step of the model, the total number of infections in each age group in each year was multiplied by an age-specific probability of progression to chronic infection [3].

### Data analysis

To estimate the number of cases imported to the United States during 1974–2008, we multiplied country-specific HBsAg prevalence by the annual number of immigrants from each country. We assumed that these prevalence rates remained constant over the 35-year time period. Results were aggregated by WHO region and by 5-year time intervals. We estimated the projected mortality due to late complications of chronic hepatitis B for cases acquired or imported during 2004–2008, assuming that cases from low-prevalence countries were likely infected during later childhood (e.g. after age 5 years) or adulthood and therefore had a 15% chance of related mortality, whereas cases from intermediate to high prevalence countries were likely infected during infancy and had a 25% chance of related mortality [4].

Ethics approval and human subjects consent were not required for this analysis, because no individualized data were collected, and no serologic testing was performed. Because human subjects were not used, the CDC IRB did not specifically waive the need for consent. The data are based on estimated HBsAg prevalence rates and overall numbers of immigrants to the United States during the stated period.

## Results

**Figure 1:**
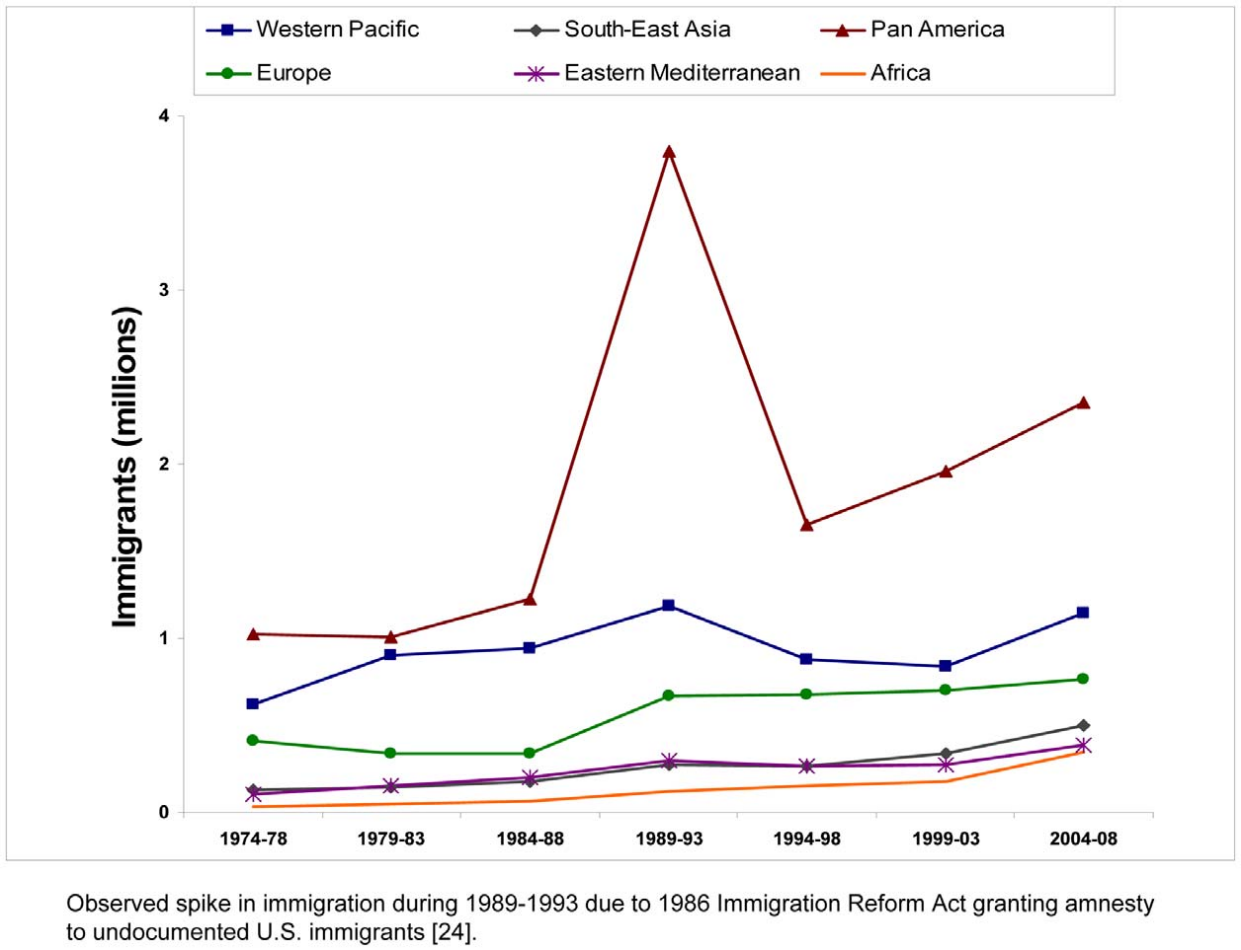
U.S. Immigration by WHO Region of Origin, 1974–2008.

During 1974–2008, 27.9 million people from 225 countries and territories immigrated to the United States, with a general upwards trend in immigration numbers (Figure 1). Of these, 63% were born in countries of intermediate or high chronic HBV infection prevalence (≥2%). Data on age at immigration were available only for 2004–2008, during which the median age category for immigrants was 30–34 years. The largest number of immigrants came from the Americas Region (13 million) and the smallest number from the African Region (940,000) (Table 1).

**Table 1:** Number of Immigrants, Estimated HBsAg prevalence, and number of imported chronic hepatitis B cases by country of birth, 1974–2008.

Birth country	No. immigrants*	(% )	Est HBsAg prevalence (%)**	Chronic hepatitis B cases	(%)
Region (no. countries)**					
Africa (48)	939,183	(3.3)	11.1	104,698	(8.0)
Americas (49)	13,201,197	(46.7)	1.6	207,800	(15.8)
Eastern Mediterranean (24)	1,695,778	(6.0)	5.0	85,565	(6.5)
Europe (58)	3,994,078	(14.1)	2.9	117,335	(8.9)
Southeast Asia (9)	1,847,292	(6.5)	4.0	73,360	(5.6)
Western Pacific (38)	6,604,083	(23.4)	11.0	724,002	(55.2)
TOTAL	28,281,611	(100.0)	4.6	1,312,760	(100.0)
**Top 10** ‡					
Philippines	1,765,203	(6.2)	10.0	176,520	(13.4)
China	1,372,025	(4.9)	12.0	164,643	(12.5)
Vietnam	1,200,863	(4.2)	12.0	144,103	(11.0)
Korea	918,505	(3.2)	12.0	110,221	(8.4)
Mexico	5,807,590	(20.5)	1.0	58,076	(4.4)
India	1,323,110	(4.7)	3.0	39,693	(3.0)
Taiwan	313,643	(1.1)	12.0	37,637	(2.9)
Dominican Republic	940,769	(3.3)	4.0	37,631	(2.8)
Haiti	532,968	(1.9)	5.0	26,648	(2.0)
Hong Kong	211,472	(0.75)	12.0	25,377	(1.9)

*From United States Department of Homeland Security, for persons obtaining legal permanent residency in United States (www.dhs.gov/files/statistics/publications/yearbook.shtm).

**World Health Organization regions. Estimated HBsAg prevalence by region is the weighted average of estimated prevalence for each country in the region.

‡ Top 10 countries by estimated number of imported chronic hepatitis B cases.

**Figure 2:**
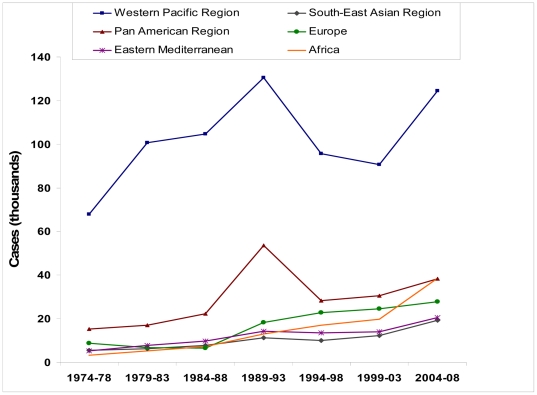
Estimated Cases of Imported Chronic HBV Infection by WHO Region of Origin, 1974–2008.

An estimated 1.3 million new cases of chronic HBV infection were imported to the United States during 1974–2008. The overall estimated prevalence of chronic HBV infection among all new immigrants over that time period was 4.6%. The general upward trend in estimated imported cases, from 105,750 cases in 1974–1978 to 268,800 cases in 2004–2008, reflects the upward trend in immigration (Figure 2). In contrast to the U.S. HBsAg prevalence of ∼0.3%, the total estimated prevalence among all immigrants to the U.S. during 2004–2008 was 4.9%.

The majority of estimated imported chronic hepatitis B cases (724,000, or 55.2%) were from the Western Pacific Region; the number of cases from the Western Pacific Region was 2.4–5.9 times greater than the second leading region for estimated cases for each 5-year time period (Figure 2). Overall, the leading three countries of birth for imported cases were the Philippines (176,520 estimated cases), China (164,640 cases), and Vietnam (144,100 cases). Together, these counties accounted for 37% of the estimated total burden of imported chronic hepatitis B.

During 1974–1978 and 2004–2008, the number of imported chronic hepatitis B cases from the African Region increased from 3,238 to 32,617. The African Region overtook the Americas Region as the region of origin for the second largest number of imported cases during 2004–2008, although the African Region still had the smallest number of U.S. immigrants (6.3%) during those years. The African country with the most U.S. immigrants during 1974–1978 was South Africa (5,835 immigrants) with an estimated HBsAg prevalence of 8%, whereas the top African country for U.S. immigrants during 2004–2008 was Ethiopia (60,720 immigrants and 11% prevalence).

**Figure 3:**
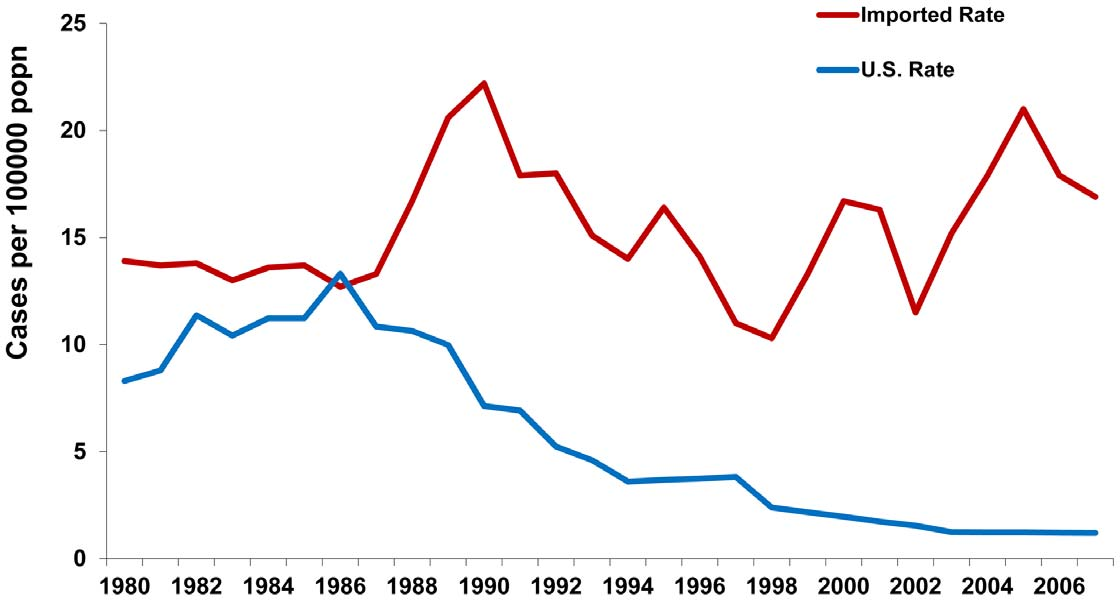
Incidence of Chronic Hepatitis B, U.S.-Acquired vs. Estimated Imported, United States, 1980–2008.

The difference between the estimated incidences of new U.S.-acquired and newly imported chronic HBV infections increased over time. In 1988, there were roughly the same number of estimated U.S.-acquired and newly imported infections (∼30,000). By 2006, the estimated number of U.S.-acquired infections had declined to 3,700, while the estimated number of newly imported infections had risen to 62,000—nearly 17 times the U.S.-acquired number (Figure 3).

During 2004–2008, there was an average of 53,800 newly imported cases of chronic HBV infection per year and 3,800 new U.S.-acquired cases per year, for a total of 57,600 cases each year. Of these, approximately 25%, or 14,000, will eventually develop fatal complications as a result of the infection if appropriate clinical management is not implemented. Most of these deaths (95.8% or 13,110) would be in immigrants with imported chronic infection.

## Discussion

Our data suggest that over the past 35 years in the United States chronic HBV infection has become a disease affecting immigrants disproportionately. This trend is the result of a substantial decline in new HBV infections acquired in the United States in the past 20 years compared to a lack of decline in the number of imported cases, such that well over 90% of new cases of chronic hepatitis B in the United States are now attributable to importation. Certain populations of immigrants, such as those from the Western Pacific and Sub-Saharan African regions, are likely at the highest risk of HBV infection prior to immigration. The national strategy for elimination of domestic transmission of HBV through immunization must take into account the burden of disease among foreign-born Americans.

This U.S. hepatitis B immunization strategy, first recommended by the Advisory Committee on Immunization Practices (ACIP) in 1991, calls for routine vaccination of infants against HBV infection beginning at birth, catch-up vaccination for older children and adolescents who have not completed the series, screening of all pregnant women to reduce transmission to the newborn, and vaccination of at-risk adults, including international travelers to regions of high or intermediate prevalence [14]. Such measures have led to a substantial decline in new U.S.-acquired cases, but cannot prevent infections acquired prior to immigration.

However, early diagnosis of chronic HBV infection can benefit immigrants and their families. Since 2008, CDC has recommended serologic screening of persons born in countries with either high or intermediate HBsAg seroprevalence [4], but immigrants are likely inconsistently screened [15]–[16]. Recent studies have demonstrated the cost-effectiveness of screening migrant populations for chronic HBV infection, both in the U.S. and in other countries [17]–[18], due to the potential for intervention in the disease process at an earlier stage [19]–[20]. Household members of persons with chronic HBV infection should be offered screening, and if susceptible, vaccination.

Voluntary screening in the United States after immigration would involve significant education and outreach efforts, in order to ensure that a large proportion of at-risk immigrants are tested. All immigrant applicants to the United States undergo a federally required medical exam as part of the application process; however, its purpose is to identify inadmissible conditions, such as active tuberculosis. Chronic HBV infection is not a condition that would preclude immigration, and therefore is not screened for at that time. The required exam may still provide an opportunity for education of applicants from intermediate and high risk countries about the disease and the importance of testing after arrival in the United States.

Such education could occur in conjunction with vaccination. Immigrants are required to receive ACIP-recommended vaccines during the exam; therefore, immigrating children receive hepatitis B vaccine [20]. However, there is no ACIP recommendation for HBV screening prior to vaccination; this may impart a false sense of immunity among vaccinated, HBV- infected individuals and discourage families from seeking screening for their child after immigration. Further, although hepatitis B vaccine is ACIP-recommended for certain groups of at-risk adults, there is no specific recommendation for vaccination of adult immigrants from intermediate or high-endemicity countries. Vaccine stakeholders should consider the current application of hepatitis B vaccine guidelines in immigrating populations, and determine whether more specific recommendations for vaccination of immigrants and others from intermediate or high-risk countries are warranted, including discussion of whether hepatitis B screening should be recommended for such individuals prior to vaccination.

Global Hepatitis B (HB) vaccine programs are another critical disease control measure that will eventually reduce chronic hepatitis B importation into the United States, a country of immigrants [21]. HB vaccine is highly effective in preventing infection [22], and as of 2008, the primary 3-dose series for infants and children has been introduced in 180 (92%) countries worldwide [23]. However, vaccine coverage is not optimal in many of these countries. For example, only a third of the countries with a high prevalence of chronic HBV infection have implemented the birth dose of HB vaccine [23]. While global immunization programs are dramatically reducing HBV infection rates in younger age groups, there would be a slower decline in the rates of chronic hepatitis B among U.S. immigrants, whose median age is currently 30–34 years [10], emphasizing the importance of screening programs targeting immigrant groups.

Our data should be interpreted with some caution. The prevalence estimates used are assumed constant for 1974–2008, while in reality, country-specific prevalence data may be changing. In addition, the data used are from a variety of sources and, in the case of countries with limited epidemiologic data, represent expert consensus rather than actual serologic prevalence. Another limitation is that country prevalence estimates may not be fully representative of the U.S. immigrant population, since immigrants may be derived from certain ethnic groups or regions which may have different HBV prevalence rates than the national average, and we did not estimate the contribution from certain groups for whom reliable population data do not exist, such as students on temporary visas or undocumented immigrants, although the estimated change in imported cases due to the 1989–1993 spike in Pan-American immigrants from low HBsAg-prevalence countries when amnesty was granted to undocumented immigrants [24]—still substantially dwarfed by cases imported from the Western Pacific region (Figure 2)—may provide an estimate of the effect from such groups. Nonetheless, even a cautious interpretation of these estimates would suggest that there is a disproportionate burden of disease in new Americans. Future studies could determine the actual, rather than estimated, HBV seroprevalence among new immigrants to the United States and assess the implementation and impact of CDC screening recommendations in reducing related morbidity and mortality among immigrants.

In conclusion, chronic hepatitis B is an important global public health concern, which in the United States disproportionately and increasingly affects the immigrant population. Because global and national immunization programs would not assist many already-infected current immigrants, earlier identification of infection is needed to prompt the initiation of appropriate disease control measures. Early screening would enable earlier referral for monitoring and therapy in the United States, and promote patient awareness and education. Such measures could reduce morbidity and mortality, as well as transmission of a common but potentially fatal infection, among new Americans.
