# Correction: The Increasing Burden of Imported Chronic Hepatitis B — United States, 1974–2008

**DOI:** 10.1371/annotation/7f73ed17-709e-4d7f-9aae-aab1f4a34985

**Published:** 2013-03-05

**Authors:** Tarissa Mitchell, Gregory L. Armstrong, Dale J. Hu, Annemarie Wasley, John A. Painter

There are changes to the second through fifth sentences of the fifth paragraph of the Discussion section. The section should be:

Immigrants are required to receive ACIP-recommended vaccines during the exam; therefore, immigrating children receive hepatitis B vaccine [21]. The ACIP currently recommends screening prior to vaccination for all persons born in high-endemicity countries.* The ACIP also recommends hepatitis B vaccine for certain groups of at-risk adults*, but makes no specific recommendation for vaccination of adult immigrants from intermediate- or high-endemicity countries.

*[CDC. A comprehensive immunization strategy to eliminate transmission of hepatitis B virus infection in the United States: recommendations of the Advisory Committee on Immunization Practices (ACIP). Part 2: immunization of adults. MMWR 2006;55(No. RR-16), p. 27-28]. 

